# Maternal and Neonatal Morbidity and Mortality Among Pregnant Women With and Without COVID-19 Infection: The INTERCOVID Multinational Cohort Study

**DOI:** 10.1001/jamapediatrics.2021.1050

**Published:** 2021-04-22

**Authors:** José Villar, Shabina Ariff, Robert B. Gunier, Ramachandran Thiruvengadam, Stephen Rauch, Alexey Kholin, Paola Roggero, Federico Prefumo, Marynéa Silva do Vale, Jorge Arturo Cardona-Perez, Nerea Maiz, Irene Cetin, Valeria Savasi, Philippe Deruelle, Sarah Rae Easter, Joanna Sichitiu, Constanza P. Soto Conti, Ernawati Ernawati, Mohak Mhatre, Jagjit Singh Teji, Becky Liu, Carola Capelli, Manuela Oberto, Laura Salazar, Michael G. Gravett, Paolo Ivo Cavoretto, Vincent Bizor Nachinab, Hadiza Galadanci, Daniel Oros, Adejumoke Idowu Ayede, Loïc Sentilhes, Babagana Bako, Mónica Savorani, Hellas Cena, Perla K. García-May, Saturday Etuk, Roberto Casale, Sherief Abd-Elsalam, Satoru Ikenoue, Muhammad Baffah Aminu, Carmen Vecciarelli, Eduardo A. Duro, Mustapha Ado Usman, Yetunde John-Akinola, Ricardo Nieto, Enrico Ferrazi, Zulfiqar A. Bhutta, Ana Langer, Stephen H. Kennedy, Aris T. Papageorghiou

**Affiliations:** 1 Nuffield Department of Women’s & Reproductive Health, University of Oxford, Oxford, United Kingdom; 2 Oxford Maternal and Perinatal Health Institute, Green Templeton College, University of Oxford, Oxford, United Kingdom; 3 Department of Paediatrics and Child Health, The Aga Khan University Hospital, Karachi, Pakistan; 4 School of Public Health, University of California, Berkeley, Berkeley; 5 Translational Health Science and Technology Institute, Faridabad, India; 6 National Medical Research Center for Obstetrics, Gynecology and Perinatology, Moscow, Russia; 7 Department of Clinical Sciences and Community Health, University of Milan, Milan, Italy; 8 Department of Woman, Child and Neonate, Fondazione IRCCS Cà Granda Ospedale Maggiore Policlinico, Milan, Italy; 9 Division of Obstetrics and Gynecology, ASST Spedali Civili di Brescia, Brescia, Italy; 10 Department of Clinical and Experimental Sciences, University of Brescia, Brescia, Italy; 11 Universidade Federal do Maranhão, São Luís, Brazil; 12 Instituto Nacional de Perinatología Isidro Espinosa de los Reyes, Mexico City, Mexico; 13 Obstetrics Department, Hospital Universitari Vall d’Hebron, Barcelona Hospital Campus, Barcelona, Spain; 14 Ospedale Vittore Buzzi Children’s Hospital, Department of BioMedical and Clinical Sciences, University of Milan, Milan, Italy; 15 Ospedale Luigi Sacco University Hospital, Department of BioMedical and Clinical Sciences, University of Milan, Milan, Italy; 16 Department of Obstetrics and Gynecology, Hôpitaux Universitaires de Strasbourg, Strasbourg, France; 17 Division of Maternal-Fetal Medicine, Brigham and Women’s Hospital, Harvard Medical School, Boston, Massachusetts; 18 Division of Critical Care Medicine, Brigham and Women’s Hospital, Harvard Medical School, Boston, Massachusetts; 19 Hôpital Universitaire Necker-Enfants Malades, AP-HP, Université de Paris, Paris, France; 20 Division Neonatología, Hospital Materno Infantil Ramón Sarda, Buenos Aires Argentina; 21 Department of Obstetrics and Gynecology, Medical Faculty, Universitas Airlangga, Surabaya, Indonesia; 22 Soetomo General Academic Hospital, Surabaya, Indonesia; 23 Tufts Medical Center, Boston, Massachusetts; 24 Ann and Robert H. Lurie Children’s Hospital of Chicago, Northwestern Feinberg School of Medicine, Chicago, Illinois; 25 St George’s University Hospitals NHS Foundation Trust, London, United Kingdom; 26 Servicio de Neonatologia del Departamento Materno Infantil del Hospital Universitario Austral, Pilar, Provincia de Buenos Aires, Argentina; 27 S.C. Obstetrics 2U, Sant’Anna Hospital, AOU Città della Salute e della scienza di Torino, Turin, Italy; 28 Fetal Medicine Unit, University College London Hospitals NHS Foundation Trust, London, United Kingdom; 29 Department of Obstetrics and Gynecology, University of Washington, Seattle; 30 Department of Global Health, University of Washington, Seattle; 31 Obstetrics and Gynaecology Department, IRCCS San Raffaele Hospital and University, Milan, Italy; 32 Fr. Thomas Alan Rooney Memorial Hospital, Asankragwa, Ghana; 33 Africa Center of Excellence for Population Health and Policy, Bayero University Kano, Kano, Nigeria; 34 Aminu Kano Teaching Hospital, Kano, Nigeria; 35 Aragon Institute of Health Research, Obstetrics Department, Hospital Clínico Universitario Lozano Blesa Zaragoza, Zaragoza, Spain; 36 College of Medicine, University of Ibadan, Ibadan, Nigeria; 37 University College Hospital, Ibadan, Nigeria; 38 Department of Obstetrics and Gynecology, Bordeaux University Hospital, Bordeaux, France; 39 Department of Obstetrics and Gynaecology, Faculty of Clinical Sciences, College of Medical Sciences, Gombe State University, Gombe, Nigeria; 40 Hospital de Moron, Moron, Provincia de Buenos Aires, Argentina; 41 Laboratory of Dietetics and Clinical Nutrition, Department of Public Health, Experimental and Forensic Medicine, University of Pavia, Pavia, Italy; 42 Clinical Nutrition and Dietetics Service, Unit of Internal Medicine and Endocrinology, ICS Maugeri IRCCS, University of Pavia, Pavia, Italy; 43 Hospital Regional Lic. Adolfo López Mateos ISSSTE, Mexico City, Mexico; 44 University of Calabar Teaching Hospital, Calabar, Nigeria; 45 Maternal and Child Department, Hospital Nacional Profesor Alejandro Posadas, Buenos Aires, Argentina; 46 Tropical Medicine and Infectious Diseases Department, Tanta University, Tanta, Egypt; 47 Department of Obstetrics and Gynecology, Keio University School of Medicine, Tokyo, Japan; 48 Department of Obstetrics and Gynaecology, Abubakar Tafawa Balewa University Teaching Hospital, Bauchi, Nigeria; 49 Sanatorio Otamendi, Ciudad de Buenos Aires, Argentina; 50 Universidad de Buenos Aires, Buenos Aires, Argentina; 51 Universidad de Moron, Moron, Argentina; 52 Department of Obstetrics and Gynaecology, Muhammad Abdullahi Wase Teaching Hospital, Kano State, Nigeria; 53 Center for Global Child Health, Hospital for Sick Children, Toronto, Ontario, Canada; 54 Women and Health Initiative, Global Health and Population Department, Harvard T.H. Chan School of Public Health, Boston, Massachusetts

## Abstract

**Importance:**

Detailed information about the association of COVID-19 with outcomes in pregnant individuals compared with not-infected pregnant individuals is much needed.

**Objective:**

To evaluate the risks associated with COVID-19 in pregnancy on maternal and neonatal outcomes compared with not-infected, concomitant pregnant individuals.

**Design, Setting, and Participants:**

In this cohort study that took place from March to October 2020, involving 43 institutions in 18 countries, 2 unmatched, consecutive, not-infected women were concomitantly enrolled immediately after each infected woman was identified, at any stage of pregnancy or delivery, and at the same level of care to minimize bias. Women and neonates were followed up until hospital discharge.

**Exposures:**

COVID-19 in pregnancy determined by laboratory confirmation of COVID-19 and/or radiological pulmonary findings or 2 or more predefined COVID-19 symptoms.

**Main Outcomes and Measures:**

The primary outcome measures were indices of (maternal and severe neonatal/perinatal) morbidity and mortality; the individual components of these indices were secondary outcomes. Models for these outcomes were adjusted for country, month entering study, maternal age, and history of morbidity.

**Results:**

A total of 706 pregnant women with COVID-19 diagnosis and 1424 pregnant women without COVID-19 diagnosis were enrolled, all with broadly similar demographic characteristics (mean [SD] age, 30.2 [6.1] years). Overweight early in pregnancy occurred in 323 women (48.6%) with COVID-19 diagnosis and 554 women (40.2%) without. Women with COVID-19 diagnosis were at higher risk for preeclampsia/eclampsia (relative risk [RR], 1.76; 95% CI, 1.27-2.43), severe infections (RR, 3.38; 95% CI, 1.63-7.01), intensive care unit admission (RR, 5.04; 95% CI, 3.13-8.10), maternal mortality (RR, 22.3; 95% CI, 2.88-172), preterm birth (RR, 1.59; 95% CI, 1.30-1.94), medically indicated preterm birth (RR, 1.97; 95% CI, 1.56-2.51), severe neonatal morbidity index (RR, 2.66; 95% CI, 1.69-4.18), and severe perinatal morbidity and mortality index (RR, 2.14; 95% CI, 1.66-2.75). Fever and shortness of breath for any duration was associated with increased risk of severe maternal complications (RR, 2.56; 95% CI, 1.92-3.40) and neonatal complications (RR, 4.97; 95% CI, 2.11-11.69). Asymptomatic women with COVID-19 diagnosis remained at higher risk only for maternal morbidity (RR, 1.24; 95% CI, 1.00-1.54) and preeclampsia (RR, 1.63; 95% CI, 1.01-2.63). Among women who tested positive (98.1% by real-time polymerase chain reaction), 54 (13%) of their neonates tested positive. Cesarean delivery (RR, 2.15; 95% CI, 1.18-3.91) but not breastfeeding (RR, 1.10; 95% CI, 0.66-1.85) was associated with increased risk for neonatal test positivity.

**Conclusions and Relevance:**

In this multinational cohort study, COVID-19 in pregnancy was associated with consistent and substantial increases in severe maternal morbidity and mortality and neonatal complications when pregnant women with and without COVID-19 diagnosis were compared. The findings should alert pregnant individuals and clinicians to implement strictly all the recommended COVID-19 preventive measures.

## Key Points

**Question:**

To what extent does COVID-19 in pregnancy alter the risks of adverse maternal and neonatal outcomes compared with pregnant individuals without COVID-19?

**Findings:**

In this multinational cohort study of 2130 pregnant women in 18 countries, women with COVID-19 diagnosis were at increased risk of a composite maternal morbidity and mortality index. Newborns of women with COVID-19 diagnosis had significantly higher severe neonatal morbidity index and severe perinatal morbidity and mortality index compared with newborns of women without COVID-19 diagnosis.

**Meaning:**

This study indicates a consistent association between pregnant individuals with COVID-19 diagnosis and higher rates of adverse outcomes, including maternal mortality, preeclampsia, and preterm birth compared with pregnant individuals without COVID-19 diagnosis.

## Teaser

This cohort study assesses the association between COVID-19 and maternal and neonatal outcomes in pregnant women with COVID-19 diagnosis compared with concomitantly enrolled pregnant women without COVID-19 diagnosis.

## Introduction

At the outset of the COVID-19 pandemic, the precise extent of the risks in pregnancy was uncertain, which was affecting pregnant individuals’ mental health.^1,2^ The lack of clarity arose because, in an early systematic review,^3^ only 4 studies that involved small numbers compared outcomes between pregnant women with and without COVID-19.^4,5,6,7^ The question is relevant because of the known deleterious effects of other coronavirus infections in pregnancy (eg, severe acute respiratory syndrome and Middle East respiratory syndrome).^8^ Therefore, the INTERGROWTH-21st Consortium conducted a prospective, longitudinal, observational study (INTERCOVID), involving 43 hospitals in 18 countries, to assess the association between COVID-19 and maternal and neonatal outcomes in pregnant women with COVID-19 diagnosis, compared with concomitantly enrolled pregnant women without COVID-19 diagnosis.

## Methods

### Study Design

The Oxford Tropical Research Ethics Committee and all local ethics committees approved the study, which did not interfere with clinical management. Informed consent (oral or written) was obtained from study participants according to local requirements, except when a waiver/exemption of such consent was granted by a local committee. We adhered to the Declaration of Helsinki^9^ and Good Clinical Practice guidelines. The study protocol, including the laboratory tests used, has been previously published.^10^

For 8 months from March 2, 2020, we enrolled women 18 years or older at any stage of pregnancy or delivery with the diagnosis of COVID-19 during the present pregnancy based on laboratory confirmation of COVID-19 and/or radiologic pulmonary findings suggestive of COVID-19^11^ or 2 or more predefined COVID-19 symptoms. A range of different real-time polymerase chain reaction and antibody tests were used at participating institutions (eBox in the Supplement). Two immediately concomitant pregnant women 18 years or older without any of those diagnostic criteria were enrolled per woman with COVID-19 diagnosis to create an unbiased sample of all pregnant women without COVID-19 diagnosis in these institutions. Women were enrolled from 43 institutions in 18 countries (Argentina, Brazil, Egypt, France, Ghana, India, Indonesia, Italy, Japan, Mexico, Nigeria, North Macedonia, Pakistan, Russia, Spain, Switzerland, UK, and the US). Data on race were not collected.

When a woman with a COVID-19 diagnosis was identified antenatally, 2 women without COVID-19 diagnosis of similar gestational age (±2 weeks) receiving standard antenatal care were enrolled that day. If not possible or if those women without COVID-19 diagnosis were lost to follow-up, we enrolled 2 women without COVID-19 diagnosis who delivered immediately after the woman with COVID-19 diagnosis. The same selection strategy was used when a woman with COVID-19 diagnosis was identified at hospital admission and delivery was likely during that admission. If a woman without COVID-19 diagnosis declined participation, the next woman was approached until 2 women without COVID-19 diagnosis were enrolled per woman with COVID-19 diagnosis. We sought confirmation from a biweekly random 10% sample that the 2 women without COVID-19 diagnosis were appropriately chosen; we excluded 5 women who had a COVID-19 diagnosis and the corresponding 10 women without a COVID-19 diagnosis, without such confirmation. Live and stillborn singleton and multiple pregnancies were included, including those with congenital anomalies. However, in keeping with reporting requirements during the pandemic,^12^ we excluded women/neonates from the final analysis if their data were already published in any comparative study with women without COVID-19 diagnosis.^5,13,14,15,16,17,18,19,20,21,22,23^

### Outcomes

The primary outcomes^24^ were 3 unweighted indices: (1) maternal morbidity and mortality index including at least 1 of the following pregnancy-related morbidities: third-trimester vaginal bleeding, pregnancy-induced hypertension, preeclampsia/eclampsia/hemolysis, elevated liver enzymes, and low platelet count (HELLP) syndrome, preterm labor, infections requiring antibiotics, or any other pregnancy-related conditions requiring treatment or referral; maternal admission to the intensive care unit (ICU); referral to a higher level of care; or death; (2) severe neonatal morbidity index (SNMI) including at least 3 of the following severe complications: bronchopulmonary dysplasia, hypoxic-ischemic encephalopathy, sepsis, anemia requiring transfusion, patent ductus arteriosus requiring treatment or surgery, intraventricular hemorrhage, and necrotizing enterocolitis or retinopathy of prematurity diagnosed before hospital discharge; and (3) severe perinatal morbidity and mortality index (SPMMI) including fetal death, at least 1 of the severe neonatal conditions listed above, admission to the neonatal ICU (NICU) for 7 days or longer, or neonatal death before hospital discharge. Secondary outcomes were each individual component of the indices described above considered as separate conditions.

Gestational age estimation was based on ultrasonography measurement of fetal crown-rump length (<14 weeks’ gestation) against the international INTERGROWTH-21st standards^25^ or, if early ultrasonography dating was not carried out, the best obstetric estimate was used based on all clinical and ultrasonography data available at the time of delivery. Newborn weight, length, and head circumference at birth were assessed against the international INTERGROWTH-21st standards.^26^

### Data Management and Statistical Analysis

We used a centrally coordinated data management system developed for the INTERGROWTH-21st Project (MedSciNet).^27^ Associations between being diagnosed as having COVID-19 and morbidity/mortality indices expressed as binary outcomes were assessed using Poisson models with a log link function and robust standard errors expressed as relative risk (RR) and 95% CI. Associations with number of days in ICU were assessed using negative binomial models with robust standard errors (expressed as an incidence rate ratio and 95% CI). We set statistical significance at *P* < .05. Models for our primary outcomes were adjusted for country, month entering study, maternal age, and history of maternal morbidity (including diabetes, thyroid, and other endocrine disorders; cardiac disease; hypertension; chronic respiratory disease; kidney disease; malaria; or tuberculosis). Models with preterm birth as an outcome were also adjusted for previous preterm birth. We plotted Kaplan-Meier curves with the percentage of women remaining pregnant by gestational age to compare the distributions between women with and without COVID-19 diagnosis, according to symptom status. We evaluated women with COVID-19 diagnosis for the primary and secondary outcomes using the women without COVID-19 diagnosis as the reference group. We further categorized women with COVID-19 diagnosis as asymptomatic or symptomatic based on type and duration of symptoms, as well as according to past maternal morbidity and normal weight or overweight to explore effect modification. We assessed the association of neonates testing positive for SARS-CoV-2 infection. In separate sensitivity analyses, we adjusted for additional potential confounders, restricted the definition of women with COVID-19 diagnosis to mothers with a positive laboratory test result, and excluded twins. We also performed separate meta-analyses for our primary outcomes of interest using the stratified results to estimate pooled effects and assess heterogeneity by country.

## Results

We enrolled 706 women with COVID-19 diagnosis, of which 656 (92.9%) had laboratory/radiological confirmation and 50 (7.1%) had more than 2 symptoms without laboratory confirmation. Of those who tested positive, almost exclusively (640 of 652 [98.1%]) by real-time polymerase chain reaction, 287 (44.0%) were asymptomatic. We also enrolled 1424 women without COVID-19 diagnosis (eFigure in the Supplement). The groups of women with and without diagnosis had similar demographic characteristics. However, 48.6% (n = 323) of the group with COVID-19 diagnosis had overweight early in pregnancy compared with 40.2% (n = 554) of the group without COVID-19 diagnosis. Women with COVID-19 diagnosis had a higher rate of recreational drug use but lower rate of smoking during the index pregnancy, higher rates of previous preterm birth, stillbirth, neonatal death, and preexisting medical conditions (eTable 1 in the Supplement).

During the index pregnancy, women with a COVID-19 diagnosis had higher rates of pregnancy-induced hypertension (RR, 1.46; 95% CI, 1.05-2.02), preeclampsia/eclampsia (RR, 1.76; 95% CI, 1.27-2.43), and infections requiring antibiotics (RR, 3.38; 95% CI, 1.63-7.01), and there was an association with a greater risk of admission to ICU/high-dependency unit (RR, 5.04; 95% CI, 3.13-8.10) and referral to a higher level of care (RR, 6.07; 95% CI, 1.23-30.01). Among all ICU admissions, women with COVID-19 diagnosis stayed 3.73 (95% CI, 2.37-5.86) days longer than women without COVID-19 diagnosis (Table 1).

**Table 1.** Pregnancy Complications, Perinatal Events, and Neonatal Morbidities Among Women With and Without COVID-19 Diagnosis and Their Newborns

Characteristic	No. (%)		Relative risk (95% CI)
	Women with COVID-19 diagnosis (n = 706)	Women without COVID-19 diagnosis (n = 1424)	
Maternal morbidity and mortality index^a^	225 (31.9)	296 (20.8)	1.54 (1.33 to 1.78)^b^
Vaginal bleeding	44 (6.2)	87 (6.1)	1.02 (0.72 to 1.46)
Pregnancy-induced hypertension	58 (8.2)	80 (5.6)	1.46 (1.05 to 2.02)
Preeclampsia/eclampsia/HELLP	59 (8.4)	63 (4.4)	1.76 (1.27 to 2.43)^b^
Hemoglobin level <10 g/dL at >27 wk gestation	130 (18.4)	228 (16.0)	1.15 (0.91 to 1.45)
Preterm labor	52 (7.4)	88 (6.2)	1.20 (0.86 to 1.68)
Infections requiring antibiotics	25 (3.6)	16 (1.1)	3.38 (1.63 to 7.01)
Admitted to ICU	59 (8.4)	23 (1.6)	5.04 (3.13 to 8.10)
Time in ICU, mean (SD), d	7.3 (7.8)	2.0 (1.7)	3.73 (2.37 to 5.86)^c^
Referred for higher dependency care	6 (0.9)	1 (0.1)	6.07 (1.23 to 30.01)
Maternal death	11 (1.6)	1 (0.1)	22.26 (2.88 to 172.11)
Fetal distress	87 (12.3)	120 (8.4)	1.70 (1.06 to 2.75)^b^
Spontaneous initiation of labor	333 (47.2)	793 (55.7)	0.85 (0.77 to 0.93)
Induced labor	157 (22.3)	320 (22.5)	0.99 (0.84 to 1.18)
Cesarean delivery	346 (49.0)	547 (38.4)	1.28 (1.16 to 1.40)^b^
Prelabor rupture of membranes	114 (16.1)	262 (18.4)	0.87 (0.71 to 1.07)
Gestational age at birth, mean (SD), wk	37.9 (3.3)	38.5 (3.1)	−0.61 (−0.90 to −0.32)^d^
Preterm birth (<37 wk gestation)	159 (22.5)	194 (13.6)	1.59 (1.30 to 1.94)^e^
Spontaneous preterm birth	27 (3.8)	66 (4.6)	0.81 (0.52 to 1.27)
Medically indicated preterm birth	133 (18.8)	127 (8.9)	1.97 (1.56 to 2.51)^e^
Birth weight, mean (SD), kg	2.96 (0.70)	3.07 (0.68)	−0.11 (−0.18 to −0.04)^d^
Male	353 (50.0)	749 (52.6)	0.95 (0.87 to 1.04)
Female	353 (50.0)	675 (47.6)	1.06 (0.96 to 1.16)
Low birth weight (<2500 g)	145 (20.5)	181 (12.7)	1.58 (1.29 to 1.94)^b^
Small for gestational age (<10th centile)^f^	97 (13.7)	181 (12.7)	1.03 (0.81 to 1.31)
Exclusive breastfeeding at discharge	378 (53.5)	953 (66.9)	0.80 (0.74 to 0.87)
Any breastfeeding at discharge	588 (83.3)	1290 (90.6)	0.92 (0.88 to 0.96)
SNMI^g^	44 (6.2)	33 (2.3)	2.66 (1.69 to 4.18)^b^
Severe perinatal morbidity and mortality index^h^	120 (17.0)	113 (7.9)	2.14 (1.66 to 2.75)^b^

Abbreviations: HELLP, hemolysis, elevated liver enzymes, low platelet count; ICU, intensive care unit; SNMI, severe neonatal morbidity index.

SI conversion factors: To convert hemoglobin to grams per liter, multiply by 10.

^a^ Maternal morbidity and mortality index includes at least 1 of the following complications during pregnancy: vaginal bleeding, pregnancy-induced hypertension, preeclampsia, eclampsia, HELLP, preterm labor, infections requiring antibiotics or maternal death, admission to ICU, or referral for higher dependency care.

^b^ Models adjusted for country, month entering study, maternal age, and history of maternal morbidity (including diabetes, thyroid and other endocrine disorders, cardiac disease, hypertension, chronic respiratory disease, kidney disease, malaria, or tuberculosis).

^c^ Incidence rate ratio and 95% CI are reported.

^d^ β and 95% CI are reported.

^e^ Models for preterm birth adjusted for history of preterm birth, country, month entering study, maternal age, and history of maternal morbidity (including diabetes, thyroid and other endocrine disorders, cardiac disease, hypertension, chronic respiratory disease, kidney disease, malaria, or tuberculosis).

^f^ Against the international INTERGROWTH-21st Newborn Size Standards.^22^

^g^ SNMI includes at least 1 of the following morbidities: bronchopulmonary dysplasia, hypoxic-ischemic encephalopathy, sepsis, anemia requiring transfusion, patent ductus arteriosus, intraventricular hemorrhage, necrotizing enterocolitis, or retinopathy of prematurity.

^h^ Severe perinatal morbidity and mortality index includes any of the morbidities listed in the SNMI or intrauterine or neonatal death or neonatal ICU stay ≥7 days.

Eleven women (1.6%) with COVID-19 diagnosis died (maternal mortality ratio, 159/10 000 births). Of these, 4 had severe preeclampsia (1 superimposed on chronic hypertension and 1 associated with cardiomyopathy); 3 of these 4 women had respiratory failure that required mechanical ventilation and the fourth woman died of a pulmonary embolism. Five women had worsening respiratory failure antenatally, 2 of whom underwent cesarean delivery and, despite intensive respiratory support, died later. The remaining 2 women developed fever, cough, and breathlessness within 7 days of an uneventful delivery and died shortly after, despite ICU care. In the group of women without COVID-19 diagnosis, there was 1 death due to preexisting liver malignant neoplasm and cirrhosis. Thus, women with COVID-19 diagnosis were 22 times more likely to die (RR, 22.3; 95% CI, 2.88-172), although the CIs were wide owing to the small numbers (Table 1).

Overall, women with COVID-19 diagnosis had a lower rate of spontaneous initiation of labor but higher cesarean delivery rate, reflecting the higher rates of pregnancy complications in this group. They also had higher RRs for preterm birth and fetal distress of 1.59 (95% CI, 1.30-1.94) and 1.70 (95% CI, 1.06-2.75), respectively. Overall, 83% of preterm births (n = 130) in women with COVID-19 diagnosis were medically indicated; hence, the increased risk in this group (RR, 1.97; 95% CI, 1.56-2.51) (Table 1). The leading indications for preterm delivery among women with COVID-19 diagnosis were preeclampsia/eclampsia/HELLP (31 [24.7%]), small for gestational age (24 [15.5%]), and fetal distress (17 [13.2%]). The proportions of spontaneous preterm birth were similar. Women with COVID-19 diagnosis had a higher low birth weight rate (RR, 1.58; 95% CI, 1.29-1.94). The rates of prelabor rupture of membranes were similar in both groups (Table 1). Fully adjusting models of our primary outcomes for all planned variables reduced the sample size owing to missing data, but affected the results minimally (eTable 2 in the Supplement).

Women with COVID-19 diagnosis delivered earlier than those without COVID-19 diagnosis after approximately 30 weeks’ gestation, with the greatest difference less than 37 weeks’ gestation. The Figure illustrates the probability of remaining pregnant after 25 weeks’ gestation for those women with COVID-19 diagnosis, stratified into those with and without symptoms. Using the log-rank test for trend of survivor curves, we observed a significant downwards trend in the gestational age distributions that progressed from women without COVID-19 diagnosis, to asymptomatic women with COVID-19 diagnosis, to symptomatic women with COVID-19 diagnosis (*P* < .001 for this trend) (Figure). In regression models, the gestational age at delivery was 0.6 weeks shorter (95% CI, −0.9 to −0.3) in all women with COVID-19 diagnosis and 0.8 weeks shorter (95% CI, −1.2 to −0.5) in symptomatic women with COVID-19 diagnosis than in women without COVID-19 diagnosis.

**Figure.**
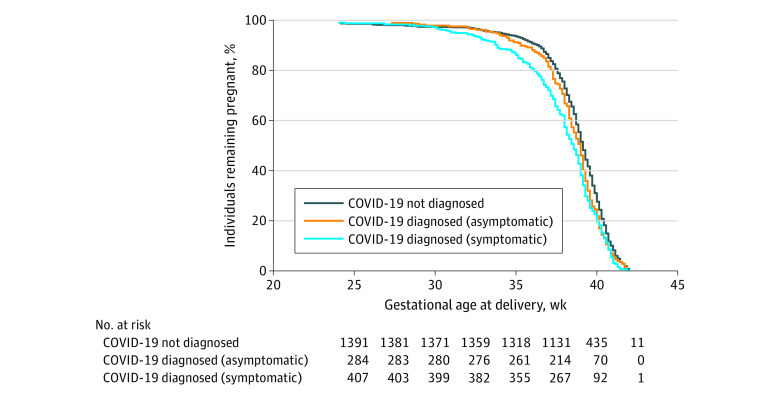
Gestational Age at Delivery Among Women With COVID-19 Diagnosis, With and Without Symptoms, and Women Without COVID-19 Diagnosis There were 1420 women without COVID-19 diagnosis (dark blue). In the group of women with COVID-19 diagnosis, 417 women were symptomatic (light blue) and 288 women were asymptomatic (orange). There was a significant trend (*P* < .001) in shorter gestational age at delivery going from women without COVID-19 diagnosis, to asymptomatic women with COVID-19 diagnosis, to symptomatic women with COVID-19 diagnosis (log-rank test for trend of survivor curves). Five women with missing data were excluded from the figure.

The risk of the SNMI among neonates of women with COVID-19 diagnosis was significantly higher (RR, 2.66; 95% CI, 1.69-4.18) than in those of women without COVID-19 diagnosis. The risk of the SPMMI was more than twice as high in the group of women with COVID-19 diagnosis (RR, 2.14; 95% CI, 1.66-2.75) (Table 1).

A total of 416 neonates born to women with COVID-19 diagnosis were tested for SARS-CoV-2 (57.1% [n = 729] of the total mother with COVID-19 diagnosis/neonate dyads); 220 (51.5%) were tested in the first 24 hours after birth and 369 (84.8%) within the first 48 hours. Of these, 54 (12.9%) tested positive. Among test-positive women with test-positive neonates, the cesarean delivery rate was 72.2% (n = 39) and among test-positive women with test-negative neonates was 47.9% (n = 173). The rate in women without COVID-19 diagnosis was 39.4% (n = 568).

In regression models, exploring factors associated with neonatal SARS-CoV-2 positivity that included gestational age at delivery, cesarean delivery, NICU stay 7 days or longer, and exclusive breastfeeding at discharge, only cesarean delivery was independently associated with the risk of a test-positive neonate (RR, 2.15; 95% CI, 1.18-3.91). Reassuringly, there was no association between exclusive breastfeeding and neonatal test positivity (RR, 1.10; 95% CI, 0.66-1.85).

eTable 3 in the Supplement shows the type, number, and prevalence of symptoms reported. Overall, the presence of any symptoms increased the association with adverse outcomes compared with the group of women without COVID-19 diagnosis. Although asymptomatic women with COVID-19 diagnosis had limited risk for most outcomes, there was still an association between the disease and preeclampsia (RR, 1.63; 95% CI, 1.01-2.63). The presence of fever and shortness of breath, separately or in combination with any symptom cluster, was markedly associated with a risk of the 3 summary indices, as well as preterm birth (Table 2).

**Table 2.** Adjusted Associations for Maternal and Perinatal Outcomes Among Women With and Without COVID-19 Diagnosis According to Symptom Status^a^

Symptom	No. (%)	RR (95% CI)				
		MMMI^b^	SNMI^c^	SPMMI^d^	Preterm birth^e^	Preeclampsia/eclampsia/HELLP
No diagnosis of COVID-19	1424 (66.9)	1 [Reference]	1 [Reference]	1 [Reference]	1 [Reference]	1 [Reference]
COVID-19						
Asymptomatic	288 (13.5)	1.24 (1.00-1.54)	1.42 (0.65-3.08)	1.08 (0.69-1.69)	0.99 (0.72-1.36)	1.63 (1.01-2.63)
Any symptom	418 (19.6)	1.76 (1.49-2.08)	3.45 (2.14-5.56)	3.09 (2.36-4.04)	2.10 (1.67-2.62)	2.00 (1.34-2.99)
Symptomatic						
With diarrhea/vomiting	48 (2.3)	1.36 (0.85-2.19)	4.66 (1.93-11.30)	2.79 (1.57-4.95)	2.76 (1.77-4.30)	0.48 (0.07-3.81)
With fever	199 (9.3)	1.89 (1.54-2.32)	4.34 (2.53-7.43)	3.81 (2.81-5.17)	2.39 (1.82-3.13)	1.82 (1.08-3.06)
With shortness of breath	89 (4.2)	2.46 (1.96-3.08)	3.88 (1.78-8.49)	3.86 (2.62-5.67)	2.88 (2.12-3.89)	2.72 (1.59-4.64)
With fever and shortness of breath	45 (2.1)	2.56 (1.92-3.40)	4.97 (2.11-11.69)	5.09 (3.30-7.86)	3.40 (2.38-4.86)	2.22 (1.06-4.64)

Abbreviations: HELLP, hemolysis, elevated liver enzymes, low platelet count; MMMI, maternal morbidity and mortality index; RR, relative risk; SNMI, severe neonatal morbidity index; SPMMI, severe perinatal morbidity and mortality index.

^a^ All models adjusted for country, month entering study, maternal age, and history of maternal morbidity (including diabetes, thyroid and other endocrine disorders, cardiac disease, hypertension, chronic respiratory disease, kidney disease, malaria, or tuberculosis).

^b^ MMMI includes at least 1 of the following complications during pregnancy: vaginal bleeding, pregnancy-induced hypertension, preeclampsia, eclampsia, HELLP, preterm labor, infections requiring antibiotics or maternal death, admission to intensive care unit, or referral for higher dependency care.

^c^ SNMI includes at least 1 of the following morbidities: bronchopulmonary dysplasia, hypoxic-ischemic encephalopathy, sepsis, anemia requiring transfusion, patent ductus arteriosus, intraventricular hemorrhage, necrotizing enterocolitis, or retinopathy of prematurity.

^d^ SPMMI includes any of the morbidities listed in the SNMI, intrauterine or neonatal death, or neonatal intensive care unit stay ≥7 days.

^e^ Models for preterm birth also adjusted for history of preterm birth.

Having shortness of breath, chest pain, and cough with fever was associated with a substantial increase in the risk for severe maternal and neonatal conditions and preterm birth. However, it appears that a longer exposure to symptoms is needed to see an associated increase in risk for preeclampsia, eg, 5 to 10 days of respiratory symptoms (RR, 2.43; 95% CI, 1.29-4.58) (eTable 4 in the Supplement).

Among those with no prepregnancy morbidities and those without overweight at their first antenatal visit, women with COVID-19 diagnosis were still at increased risk of these complications compared with women without COVID-19 diagnosis. However, for maternal outcomes, those women with COVID-19 diagnosis with prepregnancy morbidities had the highest risk in the index pregnancy, suggesting that past morbidities modify the effect of COVID-19 exposure, especially for preeclampsia/eclampsia (RR, 3.29; 95% CI, 2.03-5.33) (Table 3). Women who had overweight at the first antenatal visit and subsequently were diagnosed with COVID-19 had the highest risk for the maternal morbidity and mortality index (RR, 1.81; 95% CI, 1.48-2.21), SNMI (RR, 4.15; 95% CI, 2.15-8.01), and SPMMI and preeclampsia/eclampsia (RR, 2.62; 95% CI, 1.57-4.36), suggesting that overweight status modifies the effect of COVID-19 exposure (Table 3).

**Table 3.** Adjusted Associations Between Preexisting Maternal Morbidity or Being Overweight Prepregnancy and Maternal and Neonatal Outcomes According to COVID-19 Diagnosis^a^^,^^b^

Maternal COVID-19 diagnosis	No. (%)	RR (95% CI)				
		MMMI^c^	SNMI^d^	SPMMI^e^	Preterm birth^f^	Preeclampsia/eclampsia/HELLP
Not diagnosed						
No past morbidity	1179 (55.4)	1 [Reference]	1 [Reference]	1 [Reference]	1 [Reference]	1 [Reference]
Past morbidity	245 (11.5)	1.20 (0.92-1.54)	3.04 (1.48-6.28)	1.48 (0.95-2.29)	1.73 (1.26-2.39)	1.86 (1.11-3.12)
Diagnosed						
No past morbidity	547 (25.7)	1.57 (1.33-1.85)	4.02 (2.39-6.76)	2.35 (1.76-3.13)	1.76 (1.40-2.22)	1.88 (1.24-2.86)
Past morbidity	159 (7.5)	1.71 (1.33-2.20)	1.88 (0.74-4.73)	2.29 (1.50-3.51)	1.96 (1.41-2.73)	3.29 (2.03-5.33)
Not diagnosed						
Normal weight	823 (40.3)	1 [Reference]	1 [Reference]	1 [Reference]	1 [Reference]	1 [Reference]
Overweight	554 (27.1)	1.01 (0.81-1.24)	1.56 (0.76-3.20)	1.14 (0.78-1.67)	0.78 (0.59-1.05)	1.37 (0.82-2.30)
Diagnosed						
Normal weight	342 (16.8)	1.28 (1.03-1.58)	2.07 (0.99-4.31)	1.99 (1.38-2.88)	1.42 (1.07-1.90)	1.80 (1.06-3.07)
Overweight	323 (15.8)	1.81 (1.48-2.21)	4.15 (2.15-8.01)	2.44 (1.72-3.48)	1.43 (1.08-1.85)	2.62 (1.57-4.36)

Abbreviations: HELLP, Hemolysis, elevated liver enzymes, low platelet count; MMMI, maternal morbidity and mortality index; RR, relative risk; SNMI, severe neonatal morbidity index; SPMMI, severe perinatal morbidity and mortality index.

^a^ All models adjusted for country, month entering study, maternal age, and history of maternal morbidity (including diabetes, thyroid and other endocrine disorders, cardiac disease, hypertension, chronic respiratory disease, kidney disease, malaria, or tuberculosis).

^b^ Prepregnancy maternal morbidities included at least 1 of the following: diabetes, thyroid and other endocrine disorders, cardiac disease, hypertension, chronic respiratory disease, kidney disease, malaria, or tuberculosis.

^c^ MMMI includes at least 1 of the following complications during pregnancy: vaginal bleeding, pregnancy-induced hypertension, preeclampsia, eclampsia, HELLP, preterm labor, infections requiring antibiotics or maternal death, admission to intensive care unit, or referral for higher dependency care.

^d^ SNMI includes at least 1 of the following morbidities: bronchopulmonary dysplasia, hypoxic-ischemic encephalopathy, sepsis, anemia requiring transfusion, patent ductus arteriosus, intraventricular hemorrhage, necrotizing enterocolitis, or retinopathy of prematurity.

^e^ SPMMI includes any of the morbidities listed in the SNMI, intrauterine or neonatal death, or neonatal intensive care unit stay ≥7 days.

^f^ Models for preterm birth also adjusted for history of preterm birth.

We also compared the risk for severe neonatal complications in test-positive and test-negative neonates of women with and without COVID-19 diagnosis, the latter as a reference group. The risks for the SNMI, SPMMI, and NICU stay 7 days or longer were higher in the test-negative neonates of women with COVID-19 diagnosis. However, the test-positive neonates of women with COVID-19 diagnosis had considerably higher risk for the SPMMI and, as expected, a large increased risk for a NICU stay of 7 days or longer (Table 4).

**Table 4.** Adjusted Associations Between Maternal and Neonatal COVID-19 Diagnosis With Perinatal Morbidity and Mortality^a^

Maternal and neonatal COVID-19 diagnosis	No. (%)	RR (95% CI)		
		SNMI^b^	SPMMI^c^	NICU stay ≥7 d
Not-diagnosed mother and neonate	1462 (66.7)	1 [Reference]	1 [Reference]	1 [Reference]
Diagnosed mother but neonate not tested	313 (14.3)	1.40 (0.72-2.70)	1.68 (1.20-2.37)	1.02 (0.60-1.83)
Diagnosed mother but test-negative neonate	362 (16.5)	4.00 (2.29-6.97)	2.31 (1.69-3.17)	3.13 (2.10-4.65)
Diagnosed mother and test-positive neonate	54 (2.5)	4.13 (1.69-10.08)	3.46 (2.13-5.63)	6.03 (3.35-10.86)

Abbreviations: NICU, neonatal intensive care unit; RR, relative risk; SNMI, severe neonatal morbidity index; SPMMI, severe perinatal morbidity and mortality index.

^a^ Models adjusted for country, month entering study, maternal age, and history of maternal morbidity (including diabetes, thyroid and other endocrine disorders, cardiac disease, hypertension, chronic respiratory disease, kidney disease, malaria, or tuberculosis).

^b^ SNMI includes at least 1 of the following morbidities: bronchopulmonary dysplasia, hypoxic-ischemic encephalopathy, sepsis, anemia requiring transfusion, patent ductus arteriosus, intraventricular hemorrhage, necrotizing enterocolitis, or retinopathy of prematurity.

^c^ SPMMI includes any of the morbidities listed in the SNMI, intrauterine or neonatal death, or NICU stay ≥7 days.

In separate sensitivity analyses, adjusting for additional confounders (marital status, overweight, smoking and drug use during pregnancy), restricting mothers with COVID-19 diagnosis to laboratory-confirmed positive results and excluding twins, we found that relative risks for COVID-19–associated maternal and neonatal morbidities were similar to our main results (eTable 2 in the Supplement). Furthermore, we treated each country (all hospitals in a country pooled) as if they were separate studies in a meta-analysis. The pooled estimated RRs (95% CI) were practically identical to the unadjusted and adjusted estimations, except for the SNMI, which was increased from the unadjusted RR of 2.69 (95% CI, 1.72-4.20) to 2.91 (95% CI, 1.76-4.74). As expected, considering the variability of underlying populations and care systems, we identified some heterogeneity of RR estimates across countries in the meta-analysis for the maternal morbidity and mortality index (*I^2^* = 50.0%; *P* = .02) and SPMMI (*I^2^* = 57.4%; *P* = .005), although there was no systematic pattern among the countries.

## Discussion

We conducted a large-scale, prospective, multinational study to assess the symptoms and associations between COVID-19 in pregnancy and maternal and neonatal outcomes that included, to our knowledge for the first time, immediately concomitant pregnant women without COVID-19 diagnosis from the same populations, carefully enrolled to minimize selection bias.

We demonstrated that women with COVID-19 diagnosis, compared with those without COVID-19 diagnosis, were at substantially increased risk of severe pregnancy complications, including preeclampsia/eclampsia/HELLP syndrome, ICU admission or referral to higher level of care, and infections requiring antibiotics, as well as preterm birth and low birth weight. The risk of maternal mortality was 1.6%, ie, 22 times higher in the group of women with COVID-19 diagnosis. These deaths were concentrated in institutions from less developed regions, implying that when comprehensive ICU services are not fully available, COVID-19 in pregnancy can be lethal. Reassuringly, we also found that asymptomatic women with COVID-19 diagnosis had similar outcomes to women without COVID-19 diagnosis, except for preeclampsia.

Importantly, women with COVID-19 diagnosis, already at high risk of preeclampsia and COVID-19 because of preexisting overweight, diabetes, hypertension, and cardiac and chronic respiratory diseases,^28^ had almost 4 times greater risk of developing preeclampsia/eclampsia, which could reflect the known association with these comorbidities and/or the acute kidney damage that can occur in patients with COVID-19.^29^

Our data support reports of an association between COVID-19 and higher rates of preeclampsia/eclampsia/HELLP syndrome,^19,30^ but it is still uncertain whether COVID-19 manifests in pregnancy with a preeclampsialike syndrome or infection with SARS-CoV-2 results in an increased risk for preeclampsia. Uncertainty persists because the placentas of women with COVID-19, compared with controls, show vascular changes consistent with preeclampsia,^31^ but the state of systemic inflammation and hypercoagulability found in nonpregnant patients with severe illness and COVID-19 is also a feature of preeclampsia.^32^

It is known that in nonpregnant patients, distinct subtypes may be predictive of clinical outcomes.^33^ We found the presence of any COVID-19 symptoms was associated with increased morbidity and mortality. Specifically, severe pregnancy and neonatal complication rates were highest in women if fever and shortness of breath were present, reflecting systemic disease; their presence for 1 to 4 days was associated with severe maternal and neonatal complications. This observation should influence clinical care and referral strategies.

The risks of severe neonatal complications, including NICU stay for 7 days or longer, as well as the summary index of severe neonatal morbidity and its individual components, were also substantially higher in the group of women with COVID-19 diagnosis. The increased neonatal risk remained after adjusting for previous preterm birth and preterm birth in the index pregnancy; thus, a direct effect on the newborn from COVID-19 is likely.

Overall, our results were consistent across morbidities and mostly at an RR near or greater than 2 for maternal and neonatal outcomes, with narrow CIs excluding unity, and above 3 to 4 in several estimates. Sensitivity and stratified analyses confirmed the observed results. They are probably conservative because overall, 41% of women with COVID-19 diagnosis were asymptomatic, a subgroup with a low risk of complications. Hence, higher morbidity and mortality risk should be expected for the general pregnant population, especially in low- to middle-income countries.

We found 12.1% of neonates born to test-positive women also tested positive, a higher figure than in a recent systematic review.^34^ We speculate whether contamination at the time of cesarean delivery was responsible because the rate in this mother/neonate positive subgroup was 72.2%. Reassuringly, as SARS-CoV-2 has not been isolated from breast milk,^35^ breastfeeding was not associated with any increase in the rate of test-positive neonates.

Our results mostly reflect COVID-19 diagnosed in the third trimester. Thus, women with COVID-19 diagnosis or whose pregnancy ended earlier in pregnancy are underrepresented either because our study was exclusively hospital based or earlier infection may manifest with mild symptoms, which are either ignored or managed in primary care. Alternatively, most women might have avoided the hospital until late in pregnancy or when in labor. Clearly, the effect of COVID-19 early in pregnancy needs urgently to be studied.

### Limitations

Our study has expected limitations. Ideally, we would have collected data prospectively from all pregnancies in the participating institutions, but this was impractical because of their large number of deliveries. There was a small risk of selection bias associated with the reference group of women without COVID-19 diagnosis, despite all efforts to ensure they represented an unbiased sample of the general noninfected pregnant population. The selection of cases with COVID-19 diagnosis was affected by whether routine testing was conducted, awareness of COVID-19 symptoms particularly early in the pandemic, and the availability of test kits. Where universal testing in pregnancy has been introduced, real-time polymerase chain reaction positive rates are 0.5% to 14% in asymptomatic women.^36,37^ Hence, this group of women without COVID-19 diagnosis may have included small numbers of asymptomatic infected women (a crossover effect when women without COVID-19 diagnosis were enrolled antenatally), which would result in more conservative estimates by reducing the differences between groups. Finally, we acknowledge a risk of reporting bias relating to maternal and neonatal morbidity because women with COVID-19 diagnosis and their newborns may have been more carefully evaluated, tested, and have more events reported than in the sample of women without COVID-19 diagnosis. However, we are reassured that the results reflect a true increased risk because of our careful data monitoring and use of severe morbidity markers.

## Conclusions

In summary, in this study, COVID-19 infection during pregnancy was associated with substantial risk of morbidity and mortality in postpartum parents and their infants worldwide, compared with their not-infected pregnant counterparts, especially if the these individuals were symptomatic or have comorbidities. There is an urgent need to follow up with these parents and infants because of possible long-term health effects, including long-term COVID-19.

## Notes

Supplement. eBox. Real Time Polymerase Chain Reaction (antigen) tests and antibody tests for SARS-Cov-2 used in the participating centers eFigure. Enrollment and Covid-19 ‘exposure’ status for women participating in the INTERCOVID Study eTable 1. Baseline characteristics among women with and without Covid-19 diagnosis eTable 2. Comparisons of unadjusted and fully adjusted models for maternal and neonatal outcomes eTable 3. Distribution and duration of symptoms at the time of enrolment among women with Covid-19 diagnosis eTable 4. Associations between Covid-19 symptom duration and maternal and neonatal outcomes among women with and without COVID-19 diagnosis and their newborns eAppendix 1. Non-Author Contributors eAppendix 2. Contributors and Members of the International Study on the Effects of Covid-19 in pregnancy on maternal and newborn outcomes in the INTERGROWTH-21st global network (The INTERCOVID Study)
