# Supplementary material for: Maternal and Neonatal Morbidity and Mortality Among Pregnant Women With and Without COVID-19 Infection: The INTERCOVID Multinational Cohort Study
Source: JAMA Pediatr. 2021 Apr 22;175(8):1–10. doi: 10.1001/jamapediatrics.2021.1050 (PMC8063132; doi:10.1001/jamapediatrics.2021.1050)
Supplement: Supplement. — eBox. Real Time Polymerase Chain Reaction (antigen) tests and antibody tests for SARS-Cov-2 used in the participating centers eFigure. Enrollment and Covid-19 ‘exposure’ status for women participating in the INTERCOVID Study eTable 1. Baseline characteristics among women with and without Covid-19 diagnosis eTable 2. Comparisons of unadjusted and fully adjusted models for maternal and neonatal outcomes eTable 3. Distribution and duration of symptoms at the time of enrolment among women with Covid-19 diagnosis eTable 4. Associations between Covid-19 symptom duration and maternal and neonatal outcomes among women with and without COVID-19 diagnosis and their newborns eAppendix 1. Non-Author Contributors eAppendix 2. Contributors and Members of the International Study on the Effects of Covid-19 in pregnancy on maternal and newborn outcomes in the INTERGROWTH-21st global network (The INTERCOVID Study) [file jamapediatr-e211050-s001.pdf]

Supplemental Online Content

Villar J, Ariff S, Gunier RB, et al. Maternal and neonatal morbidity and mortality among pregnant women with and without COVID-19 infection: the INTERCOVID multinational cohort study. *JAMA Pediatr*. Published online April 22, 2021. doi:10.1001/jamapediatrics.2021.1050

eBox. Real Time Polymerase Chain Reaction (antigen) tests and antibody tests for SARS-Cov-2 used in the participating centers

eFigure. Enrollment and Covid-19 ‘exposure’ status for women participating in the INTERCOVID Study

eTable 1. Baseline characteristics among women with and without Covid-19 diagnosis

eTable 2. Comparisons of unadjusted and fully adjusted models for maternal and neonatal outcomes

eTable 3. Distribution and duration of symptoms at the time of enrolment among women with Covid-19 diagnosis

eTable 4. Associations between Covid-19 symptom duration and maternal and neonatal outcomes among women with and without COVID-19 diagnosis and their newborns

eAppendix 1. Non-Author Contributors

eAppendix 2. Contributors and Members of the International Study on the Effects of Covid-19 in pregnancy on maternal and newborn outcomes in the INTERGROWTH-21st global network (The INTERCOVID Study)

This supplemental material has been provided by the authors to give readers additional information about their work.

© 2021 Villar J et al. *JAMA Pediatr.*

eBox. Real Time Polymerase Chain Reaction (antigen) tests and antibody tests for SARS-Cov-2 used in the participating centers

Real time PCR tests	Antibody tests
• Abbott realtime SARS-Cov-2<br>• Altona diagnostics RealStar SARS-CoV-2 RT-PCR<br>• Aptima SARS-COV-2 Assay<br>• Argene SARS-CoV-2 R-gene<br>• BioFire® Respiratory 2.1 Panel with SARS-CoV-2<br>• Biozym BMS Magnetic induction cycler RT-PCR<br>• Boshpore Novel Coronovirus (2019-NCOV) detection kit<br>• Cepheid Xpert Xpress SARS-COV-2<br>• Dan Gene RT-PCR<br>• DNA-Technology Research & Production LLC Russia SARS-CoV-2/SARS-CoV Multiplex RT-PCR<br>• EUROIMMUN AG EURORealTime SARS-CoV-2<br>• GeneFinder COVID-19 Plus RealAmp Kit<br>• Hologic SARS-CoV-2 Real-time RT-PCR assay<br>• Liferiver SARS-CoV-2 E gene, N gene, ORF1ab gene<br>• Roche SARS-CoV-2 RNA PCR<br>• Sansure MA6000<br>• SD Biosensor Standard Q COVID-19 Antigen<br>• Seegene Allplex 2019-nCoV Assay<br>• Simplexa COVID-19 Direct kit<br>• TaqPath 1-Step RT-qPCR Master Mix	• Abbott SARS-Cov-2 IgG<br>• Canea rapid IgG and IgM antibodies serology kits<br>• DiaSorin Liaison SARS-CoV-2 S1/S2 IgG<br>• EUROIMMUN Anti-SARS-CoV-2 ELISA (IgG)<br>• Healgen COVID-19 Antibody Rapid Detection Kit<br>• Roche COVID-19 antibody test<br>• SD Biosensor Standard Q COVID-19 IgM/IgG Duo<br>• Shenzhen YHLO Biotech Co, Ltd SARS-CoV-2 antibodies IgM and IgG CLIA kits<br>• Vircell ELISA SAR IgM+IgA<br>• Vircell ELISA SARS-CoV-2 IgG<br>• Wondfo One Step COVID-19 rapid test<br>• Zydus Cadila IgG Kit

© 2021 Villar J et al. *JAMA Pediatr.*

eTable 1. Baseline characteristics among women with and without Covid-19 diagnosis

	Women with Covid-19 diagnosis (n=706)	Women without Covid-19 diagnosis (n=1424)
Demographic and Socioeconomic Characteristics		
Maternal age, years	30.0 ± 6.1	30.4 ± 6.1
Maternal height, cm	161.3 ± 7.6	161.3 ± 7.8
Maternal weight, kg	67.2 ± 15.8	65.2 ± 16.0
Body mass index, kg/m^2	25.8 ± 5.8	25.0 ± 5.8
Lowest hemoglobin, <27 weeks' gestation (g/dl)	11.3 ± 1.3	11.5 ± 1.4
Years of formal education	12.9 ± 4.0	12.9 ± 4.3
Worked outside the home	51.5%	51.6%
Smoker during index pregnancy	2.9%	3.8%
Alcohol (≥1 units/week)	1.8%	2.1%
Recreational drugs	2.8%	1.4%
Married or cohabiting	87.6%	88.7%
Gynecological and Obstetric History		
Hormonal contraceptives/ breastfeeding (2 months before pregnancy)	4.8%	4.6%
Fertility treatment	6.4%	6.3%
Previous pregnancy	66.8%	70.9%
Previous miscarriage	31.8%	31.1%
Previous birth	59.8%	54.6%
Previous baby <2.5kg or >4.5kg	8.7%	7.5%
Previous baby <37 weeks' gestation	7.2%	5.7%
Previous stillbirth or neonatal death	5.5%	3.3%
Pre-existing medical conditions		
Diabetes	4.7%	1.4%
Overweight (BMI ≥ 25 kg/m^2)	48.6%	40.2%
Thyroid and other endocrine	10.6%	9.2%
Cardiac disease	1.3%	1.9%
Hypertension	3.7%	2.1%
Chronic respiratory disease	3.5%	2.6%
Kidney disease	0.7%	1.1%
Malaria	1.6%	1.5%
Tuberculosis	0.4%	0.2%
Two or more of the above conditions	4.2%	2.1%

Abbreviations: BMI, body mass index.

© 2021 Villar J et al. *JAMA Pediatr.*

eTable 2. Comparisons of unadjusted and fully adjusted models for maternal and neonatal outcomes

Outcome	Unadjusted			Adjusted[a]	
	N	RR (95% CI)		N	RR (95% CI)
MMMI[b]	2101	1.53 (1.32, 1.78)		1956	1.49 (1.28, 1.74)
SNMI[c]	2131	2.69 (1.72, 4.20)		1983	2.50 (1.57, 3.99)
SPMMI[d]	2156	2.15 (1.67, 2.77)		2007	2.13 (1.64, 2.77)
Preterm birth	2094	1.66 (1.37, 2.01)		1947	1.65 (1.36, 2.01)
Preeclampsia/eclampsia/HELLP	2094	1.89 (1.34, 2.67)		1955	1.80 (1.25, 2.59)

Abbreviations: RR, relative risk; CI, confidence interval; MMMI, Maternal morbidity and mortality index; SPMMI, Severe perinatal morbidity and mortality index; SNMI, Severe neonatal morbidity index.

[a]Adjusted models include maternal age, marital status, smoking and drug use during pregnancy, overweight status, and previous history of morbidity (including diabetes, thyroid and other endocrine disorders, cardiac disease, hypertension, chronic respiratory disease, kidney disease, malaria, or tuberculosis).

[b]Maternal morbidity and mortality index includes at least one of the following complications during pregnancy: vaginal bleeding, pregnancy-induced hypertension, preeclampsia, eclampsia, HELLP (hemolysis, elevated liver enzymes, low platelet count), preterm labor, or infections requiring antibiotics; or maternal death, admission to intensive care unit, or referral for higher dependency care.

[c]Severe neonatal morbidity index includes at least one of the following morbidities: bronchopulmonary dysplasia, hypoxic-ischemic encephalopathy, sepsis, anemia requiring transfusion, patent ductus arteriosus, intraventricular hemorrhage, necrotizing enterocolitis, or retinopathy of prematurity.

[d]Severe perinatal morbidity and mortality index includes any of the morbidities listed in the Severe neonatal morbidity index; or intrauterine or neonatal death, or NICU stay ≥7 days.

© 2021 Villar J et al. *JAMA Pediatr.*

eTable 3. Distribution and duration of symptoms at the time of enrolment among women with Covid-19 diagnosis

	Covid-19 'diagnosed' women (n=706)	Symptom duration (days)
	N (%)	Median (IQR)
Chest pain	20 (2.8%)	2.5 (2 – 6)
Diarrhea/vomiting	48 (6.8%)	2 (1 – 5)
Limb or joint pain	50 (7.1%)	3 (2 – 7)
Sore throat	74 (10.5%)	3 (2 – 5)
Flu-like symptoms	80 (11.3%)	6 (2.5 – 8)
Runny nose	80 (11.3%)	3 (2 – 7)
Breathlessness	89 (12.6%)	3 (2 – 6)
Headache	91 (12.9%)	3 (2 – 5)
Tiredness/lethargy	110 (15.6%)	5 (2 – 8)
Loss of smell	120 (17.0%)	7 (3 – 10)
Fever	199 (28.2%)	3 (1 – 5)
Cough	241 (34.1%)	5 (2 – 7)
One symptom	69 (9.8%)	-
Two symptoms	135 (19.1%)	-
Three or more symptoms	214 (30.3%)	-
Asymptomatic[a]	288 (40.8%)	-

[a]Asymptomatic women with Covid-19 diagnosis.

© 2021 Villar J et al. *JAMA Pediatr.*

eTable 4. Associations between Covid-19 symptom duration and maternal and neonatal outcomes among women with and without COVID-19 diagnosis and their newborns

		MMMI[a]		SNMI[b]		SPMMI[c]		Preterm birth		Pre-eclampsia/ Eclampsia/HELLP	
	**N**	**RR**	**95% CI**	**RR**	**95% CI**	**RR**	**95% CI**	**RR**	**95% CI**	**RR**	**95% CI**
**Days with any symptom[d]**											
No Covid-19 diagnosis	1424 (66.9)	Ref		Ref		Ref		Ref		Ref	
Covid-19 diagnosis, 0 days	288 (13.5)	1.29	(1.04, 1.60)	1.35	(0.65, 2.80)	1.25	(0.82, 1.90)	1.22	(0.91, 1.63)	1.74	(1.09, 2.78)
Covid-19 diagnosis, 1-4 days	130 (6.1)	1.36	(1.01, 1.83)	4.45	(2.40, 8.25)	2.29	(1.49, 3.51)	1.90	(1.37, 2.64)	1.85	(0.97, 3.52)
Covid-19 diagnosis, 5-10 days	103 (4.8)	1.95	(1.51, 2.53)	2.93	(1.33, 6.48)	2.43	(1.57, 3.77)	1.53	(1.03, 2.30)	2.46	(1.34, 4.52)
Covid-19 diagnosis, 11+ days	185 (8.7)	1.80	(1.46, 2.23)	3.39	(1.83, 6.27)	3.29	(2.40, 4.51)	2.27	(1.76, 2.93)	1.84	(1.07, 3.17)
**Days with respiratory symptoms[d,e]**											
No Covid-19 diagnosis	1424 (66.9)	Ref		Ref		Ref		Ref		Ref	
Covid-19 diagnosis, 0 days	371 (17.4)	1.26	(1.03, 1.54)	1.88	(1.04, 3.37)	1.38	(0.97, 1.99)	1.26	(0.97, 1.64)	1.80	(1.17, 2.75)
Covid-19 diagnosis, 1-4 days	154 (7.2)	1.61	(1.26, 2.07)	3.95	(2.16, 7.22)	2.61	(1.79, 3.79)	1.82	(1.34, 2.48)	1.54	(0.81, 2.93)
Covid-19 diagnosis, 5-10 days	93 (4.4)	2.01	(1.56, 2.62)	4.49	(2.19, 9.21)	3.91	(2.70, 5.66)	2.41	(1.74, 3.32)	2.43	(1.29, 4.58)
Covid-19 diagnosis, 11+ days	88 (4.1)	2.02	(1.55, 2.64)	1.92	(0.69, 5.30)	2.67	(1.71, 4.16)	2.29	(1.63, 3.22)	2.31	(1.19, 4.50)

Abbreviations: RR, relative risk; CI, confidence interval; MMMI, Maternal morbidity and mortality index; SNMI, Severe neonatal morbidity index; SPMMI, Severe perinatal morbidity and mortality index.
[a]Maternal morbidity and mortality index includes at least one of the following complications during pregnancy: vaginal bleeding, pregnancy-induced hypertension, pre-eclampsia, eclampsia, HELLP (hemolysis, elevated liver enzymes, low platelet count), preterm labor, or infections requiring antibiotics; or maternal death, admission to intensive care unit, or referral for higher dependency care.
[b]Severe neonatal morbidity index includes at least one of the following morbidities: bronchopulmonary dysplasia, hypoxic-ischemic encephalopathy, sepsis, anemia requiring transfusion, patent ductus arteriosus, intraventricular hemorrhage, necrotizing enterocolitis, or retinopathy of prematurity.
[c]Severe perinatal morbidity and mortality index includes any of the morbidities listed in the Severe neonatal morbidity index; or intrauterine or neonatal death, or neonatal intensive care unit stay of ≥7 days.
[d]Number of days with symptoms estimated by summing the number of days mothers had each symptom.
[e] Respiratory symptoms include fever, shortness of breath, chest pain, and cough.

© 2021 Villar J et al. *JAMA Pediatr.*

eFigure. Enrollment and Covid-19 'exposure' status for women participating in the INTERCOVID Study

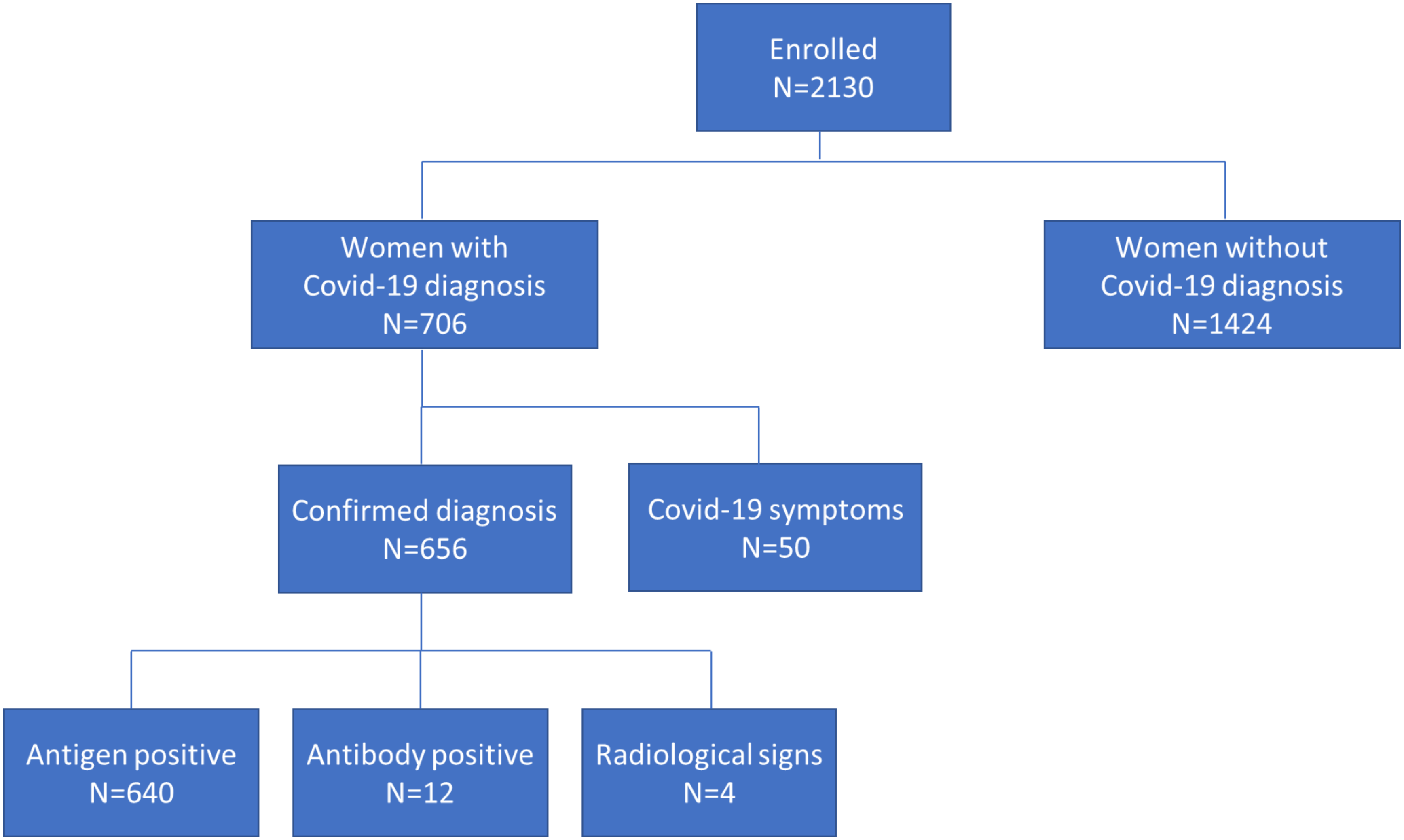

Covid-19 diagnosis was defined as a) a positive test for Covid-19 antigens or antibodies, b) radiological signs suggestive of Covid-19, or c) displaying ≥2 symptoms. The country contribution ranged from 20.4% (Italy, n=434) to 0.14% (Northern Macedonia, n=3, and Switzerland, n=3). The other sites contributed 10.4% USA (n=222), 9.8% Pakistan (n=210), 8.5% Argentina (n=180), 8.2% Mexico (n=174), 6.9% India (n=147), 6.9% France (n=146), 5.5% UK (n= 116), 5.1% Russia (n=108), 4.8% Spain (n=102), 4.4% Brazil (n=93), 3.7% Nigeria (n=78), 2.7% Indonesia (n=57), 1.7% Ghana (n=36), 0.6% Japan (n=12), and 0.4% Egypt (n=9), respectively. The median gestational age at the first ultrasound evaluation was 12 weeks (interquartile range (IQR) 10.1-16.3).

© 2021 Villar J et al. *JAMA Pediatr.*

eAppendix 1. Non-Author Contributors

Name/Highest degree	Affiliation	Contribution
Josephine Agyeman-Duah, MSc	Nuffield Department of Women's & Reproductive Health, University of Oxford, Oxford, UK.	Study Co-ordinator, Ghana
Eric Baafi, MD	Holy Family Hospital, Nkawkaw, Ghana.	Data collection Nkawkaw , Ghana
Anne Caroline Benski, MD	Hôpitaux Universitaires de Genève, Département de la Femme, de l'Enfant et de l'Adolescent, Geneva, Switzerland.	Data collection, Geneva, Switzerland
Rachel Craik, BSc	Nuffield Department of Women's & Reproductive Health, University of Oxford, Oxford, UK.	Study Co-ordinator (overall study)
Sonia Deantoni, MD	Nuffield Department of Women's & Reproductive Health, University of Oxford, Oxford, UK.<br>Oxford Maternal and Perinatal Health Institute, Green Templeton College, University of Oxford, Oxford, UK.<br>Neonatal Care Unit, Department of Public Health and Pediatrics, School of Medicine, University of Turin, Italy.	Data collection, Oxford, UK and data input (multiple sites)
Albertina Rego, PhD	Departamento de Pediatria, Faculdade Universidade Federal de Minas Gerais, Belo Horizonte, Brazil.	Study Co-ordinator, Brazil
Ken Takahashi, PhD	Department of Obstetrics and Gynecology, The Jikei University School of Medicine, Tokyo, Japan.	Data collection, Jikei, Japan
Gabriela Tavchioska, MSc	Department of Pediatrics, General Hospital Borka Taleski, Prilep, Republic of North Macedonia.	Data collection, Prilep, Republic of North Macedonia
Jim G. Thornton, MD	Division of Child Health, Obstetrics and Gynaecology, University of Nottingham, Nottingham, UK.	Literature reviews and study advisor
Adele Winsey, PhD	Nuffield Department of Women's & Reproductive Health, University of Oxford, Oxford, UK.	Study Co-ordinator (overall study)

Adele Winsey and Rachel Craik were supported by the Covid-19 Research Response Fund from the University of Oxford (Ref 0009083). Other contributors listed above did not receive any compensation.

© 2021 Villar J et al. *JAMA Pediatr.*

eAppendix 2. Contributors and Members of the International Study on the Effects of Covid-19 in pregnancy on maternal and newborn outcomes in the INTERGROWTH-21st global network (The INTERCOVID Study)

Contributors

JV, SK and ATP conceptualized and designed the INTERCOVID Study. JV, SK and ATP prepared the original protocol, with later input from AW and RC, and coordinated the project's overall undertaking with invaluable input from AL, CV, JC, BE, EF, ZAB, JR and JT. RG, SR, CC, RC and AW undertook the data management and analysis in collaboration with JV and ATP. JT and ATP undertook the ongoing literature review and interpretation of published data. The Principal Investigators at each study site implemented the protocol at their respective institutions. CC, AW and RC led the quality control of data. JV, SK and ATP wrote the manuscript with input from all co-authors. All co-authors read the manuscript and made suggestions on its content.

Members of the International Study on the Effects of Covid-19 in pregnancy on maternal and newborn outcomes in the INTERGROWTH-21st global network (The INTERCOVID Study) and its Committees.

Scientific Advisory Committee: Ana Langer (Chair), Cesar Victora, Jose Cordero, Brenda Eskenazi, Enrico Ferrazi, Zulfiqar Bhutta, Julian Robinson, Jim Thornton.

Coordinating Unit: Jose Villar, Aris Papageorghiou, Adele Winsey, Rachel Craik, Montserrat Izquierdo Renault, Stephen Kennedy, Ricardo Nieto, Albertina Rego, Josephine Agyeman-Duah.

Data Management Group: Carmen Condon, Robert Gunier, Stephen Rauch, Rachel Craik, Adele Winsey.

<u>Participating institutions and Investigators</u>

Argentina, Buenos Aires Clinica Otamendi: Carmen Vecciarell (Principal Investigator), Cristina Osio, Cecilia Baston, Marcela Volpe, Zenith Guzman, Hernan Jensen, Fernando Tami, Federico Crispin.

Argentina, Buenos Aires Hospital Materno Infantil Ramón Sarda: Ricardo Nieto and Constanza Soto Conti (co-Principal Investigators), Eugenia Luque, Jimena Melisa Vargas, Marta Isabel Lopez, Karen Zelada.

Argentina, Buenos Aires Hospital Universitario Austral: Maria Carola Capelli and Milagros Risso (co-Principal Investigators), Josefina Maria Bran.

Argentina, Buenos Aires Hospital Magdalena V de Martinez: Eduardo A Duro (Principal Investigator).

Argentina, Buenos Aires, Hospital Nacional Profesor Alejandro Posadas: Roberto Casale (Principal Investigator), Silvia Garcia, Alberto Ferreiros, Lucio Ribola, Grethelm Ferrufino, Karell Rojas, Dafne Sidiropulos, Silvana Varela.

Argentina, Buenos Aires, Hospital Municipal O. B de Lavignole: Mónica Savorani (Principal Investigator), Silvana Aguirre, Maria Rocio Tozzini.

Brazil, São Luis, Hospital Universitário da Universidade Federal do Maranhão: Marynéa Silva do Vale (Principal Investigator), Ana Claudia Garcia Marques, Patricia Franco Marques, Rebeca Aranha Arrais Santos Almeida.

Egypt, Tanta, Tanta University Hospital: Sherief Abd-Elsalam (Principal Investigator), Mohamed Elbahnasawy, Mai Khalaf, Mohamed Samir Abd El Ghafar, Eslam Saber Esmail.

France, Bordeaux, Bordeaux University Hospital: Loïc Sentilhes (Principal Investigator), Amaury Brot, Aurélien Mattuizzi, Clémence Houssin.

France, Paris, AP-HP, Hôpital Universitaire Necker-Enfants Malades: Laurent J Salomon (Principal Investigator), Joanna Sichitiu, Laurence Bussières.

© 2021 Villar J et al. *JAMA Pediatr.*

France, Strasbourg, Hôpitaux Universitaires de Strasbourg: Philippe Deruelle (Principal Investigator), Fanny De Marcillac, Mary Pontvianne, Georges-Emmanuel Roth, Charlotte Jouffrieau, Sylvain Hufschmitt-Henry, Valentine Bergthold, Mathilde Airoldi, Olivier Behra, Julie Delplanque, Coraline Schutz.

Ghana, Asankragua Fr. Thomas Alan Rooney Memorial Hospital: Vincent Bizor Nachinab (Principal Investigator), Bright Sandow, Priscilla Baffour Kyeremeh, Rita Akpene Kumi, Thomas Asechaab and Grace Hanson

Ghana, National Catholic Health Service, Directorate: George Adjei, Anita Appiah, Roberta Ama Asiedu, Ivan Essegbey.

Ghana, Nkawkaw Holy Family Hospital: Eric Baafi (Principal Investigator), Genevieve Insaidoo.

India Faridabad, **Employee's State Insurance Corporation Medical College and Hospital and affiliated Badshah Khan Civil Hospital, Haryana, India:** Ramachandran Thiruvengadam and Shinjini Bhatnagar (co-Principal Investigators), Vandita Bhartia, Mudita Wahi, Anil K. Pandey, Jagadish Chandra Sharma, Rajesh Dhiman, GARBH-Ini- DBT India Consortium for COVID Research Collaboration.

Indonesia, Surabaya, Medical Faculty Universitas Airlangga - Dr. Soetomo General Academic Hospital: Ernawati Ernawati (Principal Investigator), Hendy Hendarto, Erry Gumilar, Aditiawarman.

Italy, Brescia, Spedali Civili and University of Brescia: Federico Prefumo (Principal Investigator), Roberta Castellani, Marta Papaccio.

Italy, Milan, Fondazione IRCCS Cà Granda Ospedale Maggiore Policlinico: Paola Roggero and Enrico Ferrazzi (co-Principal Investigators), Camilla Menis, Michela Perrone, Enrico Iurlaro.

Italy, Milan, Ospedale Luigi Sacco: Valeria Savasi (Principal Investigator), Silvia Corti, Francesca Rana.

Italy, Milan, IRCCS San Raffaele Hospital and University: Paolo Ivo Cavoretto (Principal Investigator), Massimo Candiani, Giulia Bonavina.

Italy Milan, Ospedale Vittore Buzzi: Irene Cetin (Principal Investigator), Alice Zavatta, Stefania Livio.

Italy, Pavia, University of Pavia and Fondazione IRCCS Policlinico San Matteo: Hellas Cena and Rosa Maria Cerbo (co Principal Investigators), Debora Porri, Rachele De Giuseppe, Arsenio Spinillo, Annachiara Licia Scatigno, Francesca Perotti.

Italy, Turin, Ospedale Infantile Regina Margherita-Sant'Anna, Città della Salute e della Scienza di Torino: Francesca Giuliani and Manuela Oberto (co-Principal Investigators), Enrico Bertino, Pietro Gaglioti, Cristian Romolo Macchione, Francesca Carpano Maglioli.

Japan, Tokyo, The Jikei University School of Medicine: Ken Takahashi (Principal Investigator), Osamu Samura**.**

Japan, Tokyo, Keio University School of Medicine: Mamoru Tanaka and Satoru Ikenoue (co-Principal Investigators), Daigo Ochiai, Yoshifumi Kasuga, Miho Iida.

North Macedonia, Prilep, General Hospital Borka Taleski: Gabriela Tavchioska (Principal Investigator).

North Macedonia, Prilep, General Hospital Kumanovo: Aleksandra Hristova (Principal Investigator).

Mexico, Mexico City, Instituto Nacional de Perinatología Isidro Espinosa de los Reyes: Jorge Arturo Cardona-Perez (Principal Investigator), Sandra Acevedo-Gallegos, Irma Alejandra Coronado-Zarco, Brenda Ivette Frías, Addy Cecilia Helguera-Repetto, Maria José Rodriguez Sibaja, Isabel Villegas-Mota, Maria Yolotzin Valdespino.

Mexico, Mexico City, Lic. Adolfo López Mateos ISSSTE: Perla K García-May (Principal Investigator), Alberto Almanza Aguilar, Sanjuanita Gonzalez Garza, Lorena Gomez Aldape, Alejandra Loera Olvera, Paulina Soriano Cabelo.

Nigeria, Bauchi, Abubakar Tafawa Balewa University Teaching Hospital: Muhammad Baffah Aminu (Principal Investigator), Tiamiyu Ismail.

Nigeria, Calabar, University of Calabar Teaching Hospital: Saturday Etuk (Principal Investigator), Chinyere Akpanika, Komommo Okpebri, Etim Ekanem, Ubong Akpan.

© 2021 Villar J et al. *JAMA Pediatr.*

Nigeria, Gombe, Federal Teaching Hospital Gombe: Babagana Bako (Principal Investigator), Yahaya Musa Suleiman.

Nigeria, Ibadan, University College Hospital: Adejumoke Idowu Ayede and Yetunde John-Akinola (co-Principal Investigators), Oladapo OlayemI, Olufisayo Christopher Ologunore.

Nigeria, Lagos, Mainland Hospital Yaba: Abimbola Bowale (Principal Investigator), Tope Gbagada Ogunniyan.

Nigeria, Kano State, Aminu Kano Teaching Hospital: Hadiza Galadanci and Fatima Hassan-Hanga (co-Principal Investigators), Mahmoud Magashi.

Nigeria, Kano State, Muhammad Abdullahi Wase Teaching Hospital: Mustapha Ado Usman (Principal Investigator), Iman Haruna, Maryam Sulaiman, Rahila Garba, Badiyya Sayyidi, Hanifa Datti, Frank Akabudu, Abdurrahman Ali Bunawa, Amira Aminu.

Pakistan, Karachi, The Aga Khan University Hospital: Shabina Ariff (Principal Investigator), Ghulam Zainab, Lumaan Shaikh, Khalil Ahmed.

Russia, Moscow, National Medical Research Center for Obstetrics, Gynecology & Perinatology: Alexey Kholin (Principal Investigator), Irina Yakovleva, Aleksander Gus, Alexander Sencha, Roman Shmakov, Gennady Sukhikh.

Spain, Barcelona, Hospital Universitari Vall d'Hebron: Nerea Maiz (Principal Investigator), Berta Serrano, Ester del Baco, Marta Miguez, Ana Perestelo, Lidia Barberán, Cristina Tusquets, Montserrat Capell, Clementina de Antonio, Judit Gil.

Spain, Zaragoza, Hospital Clínico Universitario Lozano Blesa Zaragoza: Daniel Oros (Principal Investigator), Sara Ruiz-Martinez, Marta Fabre, Cristina Paules.

Switzerland, Geneva, Hôpitaux Universitaires de Genève: Anne Caroline Benski (Principal Investigator), Martinez de Tejada Weber, Véronique Othenin-Girard, Monia Moreau, Dominique Deletraz, David Baud.

UK, London, St George's University Hospitals NHS Foundation Trust: Aris T Papageorghiou (Principal Investigator), Becky Liu, Matthew Cauldwell, Yaa Acheampong, Danielle Hake, Sophie Robinson, Rosemary Nyamboya.

UK, London, **University College Hospitals NHS Foundation Trust:** Raffaele Napolitano (Principal Investigator), Laura Salazar, Alex Fry, Hilary Hewitt.

UK, Oxford, Oxford University Hospitals NHS Foundation Trust: Jose Villar, Stephen Kennedy, Sonia Deantoni (co-Principal Investigators), Angelika Capp, Lotoyah Carty, Kate Dixon, Yvonne Nsiah, Fenella Roseman.

USA, Boston, MA, Brigham and Women's Hospital: Sarah Rae Easter (Principal Investigator).

USA, Boston, MA, Tufts Medical Center: Mohak Mhatre (Principal Investigator), Jenny Koenig, Arome Obende.

USA, Chicago, IL, Mercy Hospital and Medical Center: Jagjit Singh Teji (Principal Investigator), Michelle Firlit.

USA, Seattle, WA, University of Washington: Michael Gravett (Principal Investigator), Lavone E Simmons.

© 2021 Villar J et al. *JAMA Pediatr.*
